# The Anesthesiologic Impact of Single-Port Robot-Assisted Partial Nephrectomy: A Tertiary Referral Comparative Analysis Between Full-Flank Transperitoneal, Retroperitoneal, and Supine Lower Anterior Access (LAA)

**DOI:** 10.3390/jpm15070306

**Published:** 2025-07-11

**Authors:** Luca Lambertini, Matteo Pacini, Paolo Polverino, Nikki R. Wilkinson, Ruben Sauer Calvo, Donato Cannoletta, Antony Angelo Pellegrino, Greta Pettenuzzo, Fabrizio Di Maida, Andrea Mari, Gabriele Bignante, Francesco Lasorsa, Alessandro Zucchi, Sergio Serni, Andrea Minervini, David B. Glick, Simone Crivellaro

**Affiliations:** 1 Department of Urology, University of Illinois Hospital & Health Sciences System, 820 S Wood St, Chicago, IL 60612, USA; 2 Unit of Oncologic Minimally-Invasive Urology and Andrology, Department of Experimental and Clinical Medicine, University of Florence, Careggi Hospital, 50134 Florence, Italy; 3 Urology Unit, Department of Translational Research and New Technologies in Medicine and Surgery, University of Pisa, 56126 Pisa, Italy; 4 Unit of Urological Robotic Surgery and Renal Transplantation, Careggi Hospital, University of Florence, 50139 Florence, Italy; 5 Department of Anesthesiology, University of Illinois at Chicago, Chicago, IL 60612, USA; 6 Unit of Urology, Division of Oncology, Urological Research Institute, IRCCS Ospedale San Raffaele, 00163 Milan, Italy; 7 Department of Urology, University of Verona, Azienda Ospedaliera Universitaria Integrata, 37126 Verona, Italy; 8 Division of Urology, Department of Oncology, San Luigi Gonzaga Hospital, University of Turin, 10043 Orbassano, Italy; 9 Urology, Andrology and Kidney Transplantation Unit, Department of Precision and Regenerative Medicine and Ionian Area, University of Bari “Aldo Moro”, 70124 Bari, Italy

**Keywords:** anesthesia, robotics, nephrectomy, renal cancer, complications

## Abstract

**Objective:** To explore the impact of supine retroperitoneal single-port robot-assisted partial nephrectomy with lower anterior access on perioperative ventilatory, cardiovascular, and pain-related outcomes compared to a cohort of patients treated with single-port robot-assisted retroperitoneal or transperitoneal partial nephrectomy with standard flank patient positioning. **Materials and Methods:** Clinical and surgical data of all consecutive patients treated with single-port robot-assisted partial nephrectomy between March 2019 and January 2024 were prospectively collected and retrospectively analyzed. Specific same-day-discharge guidelines were applied to all cases. Failed same-day discharge was defined as the presence of early (<90 days) perioperative complications or the absence of opioid-free postoperative recovery. **Results:** Overall, 105 consecutive patients treated with single-port robot-assisted partial nephrectomy were analyzed. No differences emerged in baseline features. Peak inspiratory pressure and plateau pressure changes were significantly lower in the supine retroperitoneal lower anterior access group from the time of CO_2_ insufflation throughout every 30-min operative setpoint assessment (*p* = 0.02, *p* = 0.03, and *p* = 0.02, respectively). The transperitoneal group showed significantly higher values of mean, systolic, and diastolic blood pressure compared to retroperitoneal approaches. The supine lower anterior access group also showed significantly lower non-surgical operative room time, perioperative opioid administration, and postoperative median VAS pain score. **Conclusions:** The adoption of supine lower anterior access improved perioperative ventilatory, cardiovascular, and pain-related outcomes, also optimizing operating room efficiency. Further multicenter series with longer follow-ups are still needed to validate our preliminary results.

## 1. Introduction

Lateral decubitus (LD) during open and minimally invasive partial nephrectomy represents the main patient positioning option due to its proven surgical advantages in both tans- (TP) and retroperitoneal (RP) approaches [1]. Moreover, with the constant spread of robot-assisted partial nephrectomy (RAPN) as the treatment of choice in cases of localized cT1 renal masses, a clear trend has emerged in favor of the adoption of TP approaches over their RP counterparts [2]. This factor is mainly driven by several features related to the retroperitoneal space, such as the less familiar anatomical landmarks, the narrower space in which to work, and the wider space for instrument triangulation required by multi-port (MP) robotic systems [3]. In light of this, the adoption of an RP approach might be related to several perioperative advantages such as lower estimated blood loss (EBL) and shorter operative time and length of hospital stay (LOS), particularly in cases of posterior lesions or patients with previous abdominal surgery [4,5]. Moreover, the adoption of a supine retroperitoneal approach may lead to several benefits over LD positioning, which potentially represents a time-consuming option associated with a slightly higher probability of peripheral nerve injuries [6,7,8]. In this scenario, the recent introduction of the single-port (SP) system has enriched the kidney surgery panorama with new useful access options, thus leading most surgeons to the RP approach in cases of PN [9,10]. In particular, the high flexibility of surgical instruments and the narrower space required for triangulation make it possible to embrace a supine positioning that also reaches the retroperitoneal space. Based on these assumptions, supine lower anterior access (LAA) has been developed in an attempt to conjugate retroperitoneal urinary tract surgery with supine patient positioning [11]. Although several preliminary series have validated its surgical safety and efficacy, the benefits and harms in terms of anesthesiologic perioperative features are still unexplored.

To fill this gap, we evaluated perioperative surgical, ventilatory, cardiovascular, and pain-related outcomes in a single-institution cohort of patients treated with transperitoneal, standard retroperitoneal, and supine LAA SP-RAPN.

## 2. Materials and Methods

### 2.1. Overview

The clinical and surgical data of all consecutive patients treated with single-port robot-assisted partial nephrectomy between March 2019 and January 2024 were prospectively gathered and retrospectively analyzed. All patients with clinical stage T1-T4N0 M0 amenable to PN for the detection of a renal mass were included within a prospectively maintained database. All surgical procedures were performed by a single high-volume robotic surgeon at a single tertiary referral center (UI Health, Chicago, IL, USA). Concerning the ethical standpoint, all participants were informed of the study protocol, and written consent was obtained for each patient. This study involving human participants was conducted in accordance with the ethical standards of the institutional and national research committee (approval no. IRB 2020-1428) and the 1964 Declaration of Helsinki and its later amendments or comparable ethical standards. Demographic, clinical, anesthesiologic, oncologic, and peri- and postoperative characteristics were collected using dedicated clinical software (Epic system^®^ v4.3) and gathered by a urologist and an anesthesiologist in a multi-disciplinary setting. Based on surgical approach, patients were stratified into three groups (standard retroperitoneal (RP) vs. standard transperitoneal (TP) vs. supine lower anterior access (LAA)). Throughout the study period, all of the surgical approaches were consistently available and selected based on surgeon discretion, patient-specific anatomy, and tumor characteristics. Successful same-day discharge was defined as the absence of early (<90 days) perioperative complications and the presence of opioid-free postoperative recovery.

### 2.2. Intraoperative Anesthesia Protocol

General anesthesia was induced with Propofol and maintained with Sevoflurane while Fentanyl was given as a supplementary opioid. Rocuronium was used for relaxation and was reversed with either Neostigmine or Sugammadex. For all patients included in the analysis, the neuromuscular blockade (train of four) was used. In the case of Total Intravenous Anesthesia (TIVA), unconsciousness was maintained with Propofol. In the case of a personal history of diabetes, whole blood glucose was monitored closely, and if elevated, an insulin drip was started. Esmolol was used for heart rate control, and ondansetron was used for the prevention of perioperative nausea and vomiting. Regarding lung ventilation, the same settings were used in all patients in the initial anesthesiologic steps: pressure-controlled ventilation/volume-guaranteed (PCV/VG), positive end-expiratory pressure (PEEP) 7 cm H_2_O, with respiratory rate and tidal volume adjusted as required to keep EtCO_2_ (end-tidal carbon dioxide pressure) within a normal range. Blood pressure was monitored either invasively or noninvasively every 3 min. Moreover, all patients were initially ventilated in volume-controlled mode (10 mL kg^−1^ tidal volume). Pulse-derived arterial oxygen saturation less than 97%, end-tidal CO2 partial pressure greater than 40 mm Hg, and peak inspiratory pressure greater than 36 cm H2O necessitated changes in ventilatory parameters, as deemed necessary by the anesthesiologist. If tidal volume decreased by greater than 25% as compared to baseline, pressure-controlled ventilation was begun instead. After the patient was placed in the lateral decubitus position, in the case of TP and RP procedures, PP was initiated with an IAP of 12 mmHg. Data were recorded in the supine position before and after the induction of anesthesia, while other data were recorded directly after lateral positioning and every 30 min after CO_2_ insufflation. Two additional periods of separate measurement were made at the beginning and end of insufflation.

### 2.3. Surgical Technique

All surgical procedures were performed with the Intuitive daVinci SP system^®^. The surgical approach was defined according to the surgeon’s preference and patient-related features. Insufflation was performed in all cases with a mean 12 mmHg pressure. In the case of the TP approach, patients were placed in a modified flank position at a 45-degree angle, while in the case of standard RP, patients were positioned in the full-flank position. As previously described [11], in the case of LAA, the patients were placed in the supine position, with a rubber roll under the ipsilateral flank to create a minimal body tilt. Surgical incisions were carried out differently according to the access type: in the case of a standard TP procedure, a 3 cm single incision was made laterally to the umbilicus, while a 3 cm single incision was made between the 12th rib and the superior iliac spine for the RP approach. When LAA was performed, a single 3 cm incision was carried out at the approximate McBurney’s point, 3 cm medial and 3 cm anterior to the superior iliac spine. After subcutaneous tissue dissection, the anterior fascia and the abdominal muscles (external, internal, oblique, and transversalis muscles) were spread, and the retroperitoneal space was exposed and, with a finger, developed. An 8 mm AirSeal assistant port (ConMed Corp., Utica, NY, USA) was placed in “SideCar” mode by using the same skin incision but a different fascia incision. With this approach, the SP robotic camera was typically inserted at the 6 o’clock position. Pneumoperitoneum was required for the TP approach, while retroperitoneal insufflation was used for the RP and LAA approaches. In the TP approach, the first step was to mobilize the colon to access the retroperitoneal space. This step was unnecessary for the RP and LAA approaches because of the direct exposure to the renal hilum. After identifying and isolating the renal hilum for all three approaches, the next step involved exposing the kidney and identifying the renal mass, utilizing an intraoperative ultrasound. Tumor resection was performed after clamping the renal artery and, in some cases, the renal vein, while the clamp-off technique was used in selected cases.

### 2.4. Perioperative Clinical Pathway

General anesthesia and the postoperative early-discharge assessment were performed according to the specific same-day discharge (SDD) guidelines [12]. In particular, in all patients, narcotics and polypharmacy were avoided in the case of a VAS score <1 to reduce the risk of delirium and postoperative nausea and vomiting occurrence. The intraoperative anesthesiology protocol included the use of Sevoflurane or TIVA (in this case, unconsciousness was maintained with Propofol). The perioperative pain assessment was performed at 15, 30, 60, 90, and 120 min after surgery and then at discharge. The mean value was then reported for statistical purposes. In the case of persistent pain, VAS ≥ 2 occurrence, patients were managed with nonopioid oral analgesics. In the case of patients unfit for the SDD pathway, the perioperative care protocol was applied, and inpatient ward placement was performed. Postoperative SDD eligibility was defined according to several key elements: (1) return to baseline respiratory functions, (2) adequate urine output, and (3) toleration of oral nutrition. Laboratory investigations were performed prior to discharge only if driven by clinical suspicion or if the patient had relevant preoperative comorbidities. At discharge, patients were given detailed instructions by a nurse practitioner, including expectations for recovery. Contact information was also provided with instructions to call regarding any concerns before visiting the emergency department. Moreover, to be eligible for SDD, most patients were required to have a caregiver able to drive them home and be present with them for the first 24 to 48 h after surgery. Follow-up routine visits were carried out at approximately 1 week and 1–3 months after surgery and then according to the AUA guidelines for renal carcinoma [13].

### 2.5. Statistical Analysis

Statistical analyses were performed and reported following established guidelines [14]. For statistical purposes, independent variables included all patient- and tumor-related data available in our institutional database. First, descriptive statistics were obtained, reporting means (and standard deviations, SD) for continuous variables and frequencies and proportions for categorical variables, as appropriate. Continuous variables were compared using the Student’s *t* test (categorized into three variable comparisons) and the Kruskal-Wallis rank-sum test, based on their normal or non-normal distribution, respectively (the normality of the variables’ distribution was tested using the Kolmogorov–Smirnov test). We statistically analyzed baseline characteristics and peri- and postoperative outcomes, comparing the TP, RP, and LAA approaches. Multivariable logistic regression analysis was used to test the probability of successful same-day discharge, adjusting for surgical approach, ASA score, CCI, and BMI. All statistical analyses were performed using R software (version 4.3.2). Transparent Reporting of a multivariable prediction model for Individual Prognosis Or Diagnosis (TRIPOD) and STrengthening the Reporting of OBservational studies in Epidemiology (STROBE) were used to report multivariable predictions and observational comparative analyses, respectively.

## 3. Results

**Figure 1:**
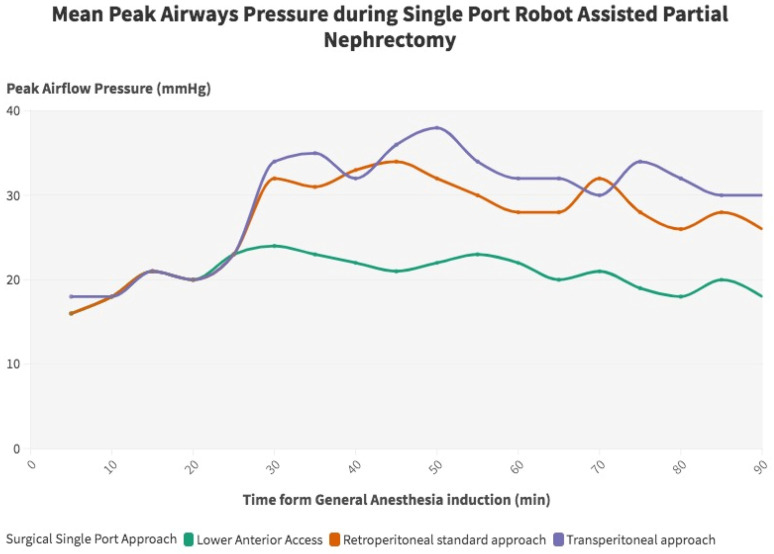
Peak airway flow pressure during single-port robot-assisted partial nephrectomy.

**Figure 2:**
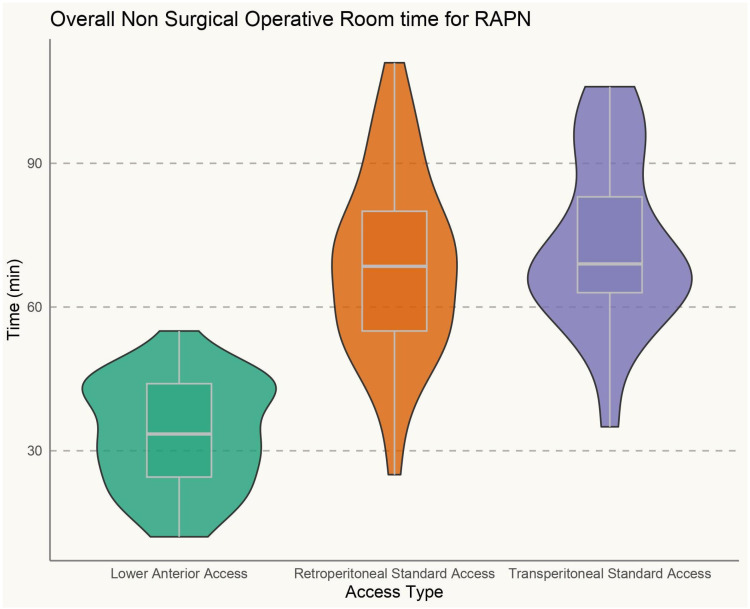
Non-surgical operative room time during single-port robot-assisted partial nephrectomy.

Overall, the clinical and surgical data of 105 consecutive patients treated with single-port RAPN at a single tertiary referral center were prospectively collected and retrospectively analyzed. Among them, 38 (36.2%) were treated with supine retroperitoneal SP RAPN with LAA, while 42 (40%) and 25 (23.8%) patients underwent SP retroperitoneal and transperitoneal PN with standard LD, respectively. Baseline characteristics are outlined in Table 1. The median recoded age was 59 (IQR 51–69), while the median BMI was 29.86 kg/m^2^ (IQR 25.2–37.8), 30.12 kg/m^2^ (IQR 26.1–36.6), and 31.30 kg/m^2^ (IQR 25.1–38.3) in the supine LAA SP-RAPN, standard retroperitoneal SP-RAPN, and transperitoneal SP-RAPN groups, respectively (*p* = 0.9). No differences emerged in baseline patient complexity with a median CCI score of 3 (IQR 2–4), a slightly lower although not significant median ASA score in the transperitoneal SP-RAPN group and comparable rates of Chronic Obstructive Pulmonary Disease (COPD) (*p* = 0.7, *p* = 0.1, and *p* = 0.7, respectively). As regards tumor features, no significant differences were recorded between groups in terms median RENAL Nephrometry Score (6 [IQR 5–7] vs. 6 [IQR 4–6] vs. 5 [IQR 4–7], *p* = 0.8), as well as in terms of clinical T stage (*p* = 0.4), while a significantly higher rate of posterior tumors was recorded within the standard retroperitoneal SP-RAPN group (*p* = 0.04). We further analyzed tumor locations across the three groups. In particular, the LAA group included 58% anterior, 26% posterior, 8% hilar, and 8% upper pole tumors, the retroperitoneal group included 39% anterior, 49% posterior, 8% hilar, and 4% upper pole tumors, while the transperitoneal group included 52% anterior, 32% posterior, 12% hilar, and 4% upper pole tumors. We also performed a component-level analysis of the RENAL nephrometry score, with tumor size, proximity to the collecting system, and exophytic/endophytic properties being comparable across the three groups. Perioperative ventilatory characteristics are outlined in Table 2. When it comes to intraoperative ventilatory outcomes, peak inspiratory pressure, plateau pressure changes, and mean PIP value were significantly lower in the supine retroperitoneal LAA group compared to the standard retroperitoneal and transperitoneal SP-RAPN groups from the time of CO_2_ insufflation throughout all 30 min assessments, but not during induction (*p* = 0.2). Figure 1. End-tidal carbon dioxide (ETCO_2_) and SpO_2_ were comparable across groups. In terms of hemodynamic features, the transperitoneal SP-RAPN group showed significantly higher values of mean, systolic, and diastolic blood pressure compared to the retroperitoneal approaches (*p* = 0.03, *p* = 0.04, and *p* = 0.04, respectively). Intraoperative opioid administration was significantly lower in the supine retroperitoneal LAA group (10 [IQR 6–12] vs. 14 [IQR 12–18] and 14 [IQR 12–20] in the standard retroperitoneal SP-RAPN and transperitoneal SP-RAPN groups, respectively, *p* = 0.02). The supine retroperitoneal LAA group showed a significantly shorter median non-surgical operative room time (41 [IQR 35–62] vs. 54 [IQR 42–73] and 54 [IQR 45–76] in the standard retroperitoneal SP-RAPN and transperitoneal SP-RAPN groups, respectively, *p* = 0.003), Figure 2, as well as a shorter console time (*p* = 0.01). Conversely, no differences emerged in terms of intraoperative EBL, ischemia time, and intraoperative complications (*p* = 0.3, *p* = 0.2, and *p* = 0.9, respectively). Overall, postoperative median VAS pain score was significantly lower in the LAA group (*p* = 0.001) with less frequent occurrence of hip and back pain as compared to standard-access groups (*p* = 0.013) and significantly reduced hospital-administered intake of opioids during POD 0–1 (*p* = 0.001). Successful same-day discharge was achieved more often in patients treated with SP-RAPN with LAA as compared to the other groups, with a rate of 84% (*p* = 0.001). In terms of mid-term surgical and functional outcomes, after a median follow-up of 16 months (IQR 6–24), no differences were recorded in terms of 90-day re-admission or re-medication rate or significant renal function loss (*p* = 0.7, *p* = 0.6, and *p* = 0.7, respectively). Failure to meet the SDD criteria occurred in 68%, 38%, and 13.2% of patients treated with the transperitoneal, standard retroperitoneal, or supine LAA approach, respectively (*p* = 0.001), Table 3. In the multivariable logistic regression models, the transperitoneal and standard retroperitoneal SP groups were confirmed as independent predictors of failure to meet SSD criteria (OR, 2.51 [95% confidence interval [CI] 1.26–4.26], *p* = 0.01) and (OR 1.71 [95% CI 1.02–2.56], *p* = 0.02), respectively. Moreover, CCI > 3 (OR, 1.67 [95% confidence interval [CI] 1.04–3.49], *p* = 0.02), BMI > 30 kg/m^2^ (OR, 1.91 [95% confidence interval [CI] 1.18–3.88], *p* = 0.04) and preoperative anticoagulant therapy (OR, 2.02 [95% confidence interval [CI] 1.98–4.58], *p* = 0.01) were also confirmed as independently associated with SSD failure. 

## 4. Discussion

During the constant paradigm shift of nephron-sparing surgery toward minimally invasive surgery, lateral flank patient positioning has always played a pivotal role, representing the main surgical option for both transperitoneal and retroperitoneal procedures [15,16]. This factor is mainly driven by its surgical advantages over the supine approach, ranging from early retroperitoneal open surgery experience to the widespread use of laparoscopic and multi-port robotic PN [17]. Nonetheless, the lateral decubitus position may also represent a time-consuming placement choice that is possibly associated with position-related physiological changes [18]. In this context, the advent of the daVinci Single-Port (SP) robotic system has opened up the field to novel access options, enabling better instrument triangulation and dexterity in narrow areas. In particular, the LAA was developed to standardize supine SP access to the retroperitoneal space, showing promising results in terms of surgical feasibility and efficacy in the upper urinary tract [11]. Conversely, an in-depth evaluation of its benefits and disadvantages in terms of perioperative ventilatory, cardiovascular, and pain-related outcomes is still lacking in the current literature.

To the best of our knowledge, our study represents the first series evaluating the perioperative impact of supine retroperitoneal RAPN performed with SP-LAA on anesthesiologic features compared to a cohort of patients treated with transperitoneal and retroperitoneal SP-RAPN with LD.

The first key point of our research is that the adoption of the LAA approach significantly improved intraoperative ventilatory and cardiovascular outcomes as compared to the procedures performed with standard LD. In particular, in terms of ventilatory features, exploiting supine positioning was associated with significantly lower values of peak inspiratory pressure in both peak and plateau items in all of the intraoperative assessments, excluding the post-induction one. These findings are possibly driven by the negative impact of flank positioning on respiratory function during minimally invasive surgery [18]. Indeed, given the lack of evidence in the current literature, the authors of several series have reported a significant reduction in pulmonary compliance, tidal volume, and vital capacity in the case of lateral flank positioning, thus enhancing ventilation–perfusion mismatch occurrence in the case of patients with respiratory dysfunction [7]. Conversely, Nadu et al. [19] found no position-related differences in terms of intraoperative ventilatory outcomes in an early series of patients treated with laparoscopic transperitoneal vs. retroperitoneal radical nephrectomy. Nevertheless, as widely stated, the different CO_2_ diffusion space between transperitoneal and retroperitoneal procedures influences intraoperative respiratory parameters, thus preventing definitive conclusions from being drawn regarding the real impact of patient positioning in this regard. To prevent approach-related biases, in our series, the main improvements in intraoperative pulmonary function were found in comparing LAA with both transperitoneal and retroperitoneal PN. From a cardiovascular standpoint, slightly higher systolic, diastolic, and mean blood pressure values were recorded only in the case of transperitoneal surgery, thus suggesting a potential benefit of retroperitoneal surgery in this scenario. Nevertheless, given the limited statistical power of the analysis, no definitive conclusions can be drawn. Our results indicated that TP surgery was associated with higher intraoperative systolic, diastolic, and mean blood pressure values as compared to the retroperitoneal approaches. However, we acknowledge that the lack of significant differences between LAA and standard retroperitoneal approaches suggests that the cardiovascular benefit may be intrinsic to the retroperitoneal route rather than exclusive to the LAA technique. This finding warrants further focused investigation in future studies. Similarly, the current literature acknowledges a major impact of CO_2_ pneumoperitoneum on hemodynamic features such as heart ratio, mean arterial pressure, and central venous pressure, mainly due to IVC compression and bilateral diaphragmatic displacement [20].

These findings constitute an even stronger argument, considering that our study population was mainly composed of complex patients with a high prevalence of systemic issues such as obesity, hypertension, and diabetes. In this scenario, minimizing intraoperative cardiopulmonary distress can play a pivotal role in increasing the procedure’s safety, thus widening the benchmarks of surgical eligibility.

The second key finding of our research is the lower perioperative pain score recorded in the LAA group, also associated with a significant reduction in intra- and postoperative opioid administration. In this regard, the authors of several series have outlined significantly reduced perioperative pain occurrence in the case of retroperitoneal surgery compared to transperitoneal surgery [2,21], possibly due to the lack of peritoneal irritation. Moreover, in our series, flank lateral positioning was associated with a higher incidence of hip and back pain, probably also due to the high rate of obese patients included in the analysis. Undoubtedly, although the access-related learning curve has not been explored in the current literature, cautious preperitoneal fat dissection as well as potential peritoneal breach is still a burden on preliminary access experiences. As regards skin and fascial incision, LAA through McBurney’s point allows for careful preservation of neighboring nerve structures, such as the ilioinguinal and iliohypogastric nerves, avoiding immediate and/or chronic neuropathic pain occurrence [22,23,24].

Third, a significantly shorter non-surgical operating room (OR) time and overall operative time were found among the LAA group compared to the RP and TP groups. This factor is mainly driven by the avoidance of anesthesiologic issues during lateral positioning, which may occur particularly when dealing with patients with a BMI > 30 kg/m^2^. In this regard, LAA improves the time efficiency of OR time, also possibly reducing peripheral nerve and soft-tissue injuries.

Finally, CCI ≥ 3, BMI ≥ 30 kg/m^2^, and surgical approach (TP and standard RP) were confirmed as independent predictors of failure to achieve SDD, confirming the feasibility of LAA in performing RAPN in an outpatient setting.

To the best of our knowledge, this is the first comparative study assessing the impact of supine SP RAPN with LAA on perioperative ventilatory, cardiovascular, and pain-related outcomes compared to standard TP and RP SP-RAPN. Nevertheless, this study is not devoid of limitations. First, the single-center, single-surgeon, and retrospective nature of the analysis may have led to non-negligible biases. In particular, given the high surgical volume (>1500 robotic cases), the surgeon’s learning curve may have influenced the perioperative outcomes as well as the overall operative time, thus limiting the clinical value of the reported results. Moreover, although a standardized postoperative SSD pathway was applied for all cases, the inter-personal variability in clinical management may have influenced the rates of SSD. Secondly, the small sample size may reduce the reproducibility of the reported results, also preventing a more weighted comparative analysis. To date, to the best of our knowledge, this constitutes the largest series of SP PN in the current literature. Finally, different anesthesiologists belonging to the same team performed the procedures, thus introducing higher inter-personal variability in perioperative management, particularly regarding the presence of patients treated with Total Intravenous Anesthesia. Nonetheless, the same institutional guidelines were applied in all procedures, and the baseline anesthesiology features were comparable among groups. In conclusion, more multi-center series with longer follow-up and larger sample sizes are still warranted to assess our preliminary outcomes.

## 5. Conclusions

The adoption of supine lower anterior access improved perioperative ventilatory, cardiovascular, and pain-related outcomes and also optimized operative room efficiency. Further multicenter series with longer follow-up are still needed to validate our preliminary results.

## Figures and Tables

**Table 1:** Baseline demographic characteristics and preoperative features.

Characteristic		Overall, N = 105 ^1^	Surgical Approach			*p*-Value ^2^
			Transperitoneal Access N = 25 ^1^	Retroperitoneal Flank Access N = 42 ^1^	Lower Anterior Access N = 38 ^1^	
**Age (years), median (IQR)**		59 (1–69)	60 (47–66)	61.50 (52.5–69.8)	58.00 (52–64.8)	0.6
**BMI (kg/m^2^), median (IQR)**		30.14 (26.3–36.5)	31.30 (25.1–38.3)	30.12 (26.1–36.6)	29.86 (25.2–37.8)	0.9
**Male gender, n (%)**		59 (56%)	16 (64%)	25 (60%)	18 (47%)	0.4
**CCI, median (IQR)**		3 (2–4)	3 (2–4)	3 (2–4)	3 (2–4)	0.7
**Substance abuse, n (%)**		17 (16%)	5 (20%)	6 (14%)	6 (16%)	0.8
**Hypertension, n (%)**		73 (70%)	17 (68%)	30 (71%)	26 (68%)	0.9
**Hypercholesterolemia, n (%)**		37 (35%)	5 (20%)	16 (38%)	16 (42%)	0.2
**COPD, n (%)**		32 (30%)	6 (24%)	14 (33%)	12 (32%)	0.7
**Diabetes, n (%)**		33 (31%)	9 (36%)	15 (36%)	9 (24%)	0.4
**Obesity, n (%)**		55 (52%)	14 (56%)	22 (52%)	19 (50%)	0.9
**Anticoagulant therapy, n (%)**		20 (19%)	8 (32%)	7 (17%)	5 (13%)	0.2
**ASA, median (IQR)**		3 (2–4)	2 (2–3)	3(2–3)	3 (2–4)	0.10
**Abdominal surgery, n (%)**		45 (43%)	11 (44%)	18 (43%)	16 (42%)	>0.9
**Preoperative Hb (g/dL), median (IQR)**		13.0 (9.2–16.8)	13.2 (9.8–16.7)	13.0 (10.1–15.2)	12.9 (10.6–14.2)	0.6
**Preoperative eGFR (mL/min/1.72 m^2^), median (IQR)**		76.0 (57–97.2)	78.0 (47.8–86.6)	76.8 (45.2–81.4)	71.0 (58.5–87.2)	0.9
**Clinical T stage, n (%)**						0.4
	cT1a	75 (71.4)	17 (76.0)	31 (76.2)	27 (73.6)	
	cT1b	20 (22.9)	4 (16.0)	8 (19.0)	8 (21.1)	
	cT2a	8 (5.7)	2 (8.0)	3 (4.8)	3 (5.3)	
**Renal nephrometry score, median (IQR)**		7 (4–7)	6 (4–7)	7 (5–8)	7 (6–8)	0.07

Legend: BMI: Body Mass Index, CCI: Charlson Comorbidity Index. ^1^ Median (IQR); n/N (%). ^2^ Kruskal–Wallis rank-sum test; Pearson’s Chi-squared test; Fisher’s exact test.

**Table 2:** Perioperative ventilatory, cardiovascular, and pain-related features.

Characteristic		Overall, N = 105 ^1^	Surgical Approach			*p*-Value ^2^
			Transperitoneal Access N = 25 ^1^	Retroperitoneal Flank Access N = 42 ^1^	Lower Anterior Access N = 38 ^1^	
**Peak inspiratory pressure (cm H_2_O), median (IQR)**		26 (22–36)	28 (24–34)	26.0 (22–34)	20 (16–26)	**0.03**
	Induction		16 (10–20)	16 (9–16)	14 (9–16)	0.2
	30 min		34 (24–36)	32 (24–34)	24 (14–24)	**0.02**
	60 min		32 (24–34)	28 (26–32)	22 (14–24)	**0.03**
	90 min		30 (24–34)	26 (26–34)	18 (14–24)	**0.04**
	120 min		30 (26–38)	28 (24–28)	18 (14–26)	**0.03**
	150 min		32 (24–36)	28 (22–30)	20 (16–24)	**0.04**
**EtCO_2_, median (IQR)**		37 (31–42)	37 (30–44)	37 (32–39)	34 (28–38)	0.2
**SpO_2_, median (IQR)**		99 (97–100)	98 (96–99)	99 (98–100)	99 (98–100)	0.7
**Systolic blood pressure (mmHg), median (IQR)**		123 (98–136)	136 (102–146)	123 (96–136)	118 (96–130)	**0.03**
**Diastolic blood pressure (mmHg), median (IQR)**		71 (50–90)	82 (64–100)	70.50 (52–88)	67.50 (54–92)	**0.04**
**Mean blood pressure (mmHg), median (IQR)**		97 (74–120)	105 (82–123)	95 (73–115)	93 (72–118)	**0.04**
Intraoperative opioid administration *, median (IQR)		12 (10–18)	14 (12–20)	14 (12–18)	10 (6–12)	**0.023**
**Postoperative pain score, median (IQR)**		4 (3–6)	5 (3–7)	5 (4–6)	3 (2–4)	**0.001**
**Opioid use on 0 or 1 PO day, n (%)**		62 (59.0)	18 (72.1)	31 (73.8)	13 (34.2)	**0.001**
**Total Intravenous Anesthesia (TIVA), n (%)**		5 (4.8)	1 (4)	2 (4.8)	2 (5.2)	0.2

Legend: EtCO_2_: End-tidal carbon dioxide; PO: postoperative, morphine equivalent. ^1^ Median (IQR); n/N (%). ^2^ Kruskal–Wallis rank-sum test; Fisher’s exact test. * Morphine equivalent.

**Table 3:** Perioperative surgical features and mid-term functional assessment.

Characteristic	Overall, N = 105 ^1^	Surgical Approach			*p*-Value ^2^
		Transperitoneal Access N = 25 ^1^	Retroperitoneal Flank Access N = 42 ^1^	Lower Anterior Access N = 38 ^1^	
**Operative time (min), median (IQR)**	184 (81)	218 (75)	190 (95)	169 (45)	**0.012**
**Non-surgical OR time (min), median (IQR)**	51 (39–71)	54 (45–76)	54 (42–73)	41 (35–62)	**0.003**
**EBL (cc), median (IQR)**	50 (42.5–200)	50 (35–200)	70 (50–100)	90 (50–150)	0.2
**Ischemia time (min), median (IQR)**	19 (17–24)	21 (15–28)	22 (14–32)	21.5 (19.2–31)	0.07
**Intraoperative complications, n (%)**	4 (3.8)	3 (12)	1 (2.4)	0 (0)	0.057
**Drainage placement, n (%)**	21(20)	13 (52)	8 (19)	0 (0)	**<0.001**
**Successful same day discharge, n (%)**	58 (55)	4 (16)	22 (52)	32 (84)	**<0.001**
**Postoperative complications, n (%)**	9 (8.6)	4 (16)	4 (9.5)	1 (2.6)	0.2
**Length of hospital stay (days), median (IQR)**	1 (0–2)	1 (1–2)	0 (0–2)	0 (0–1)	**<0.001**
**Failure to meet SSD criteria, n (%)**	27 (25.7)	17 (68)	16 (38)	5 (13.2)	**<0.001**
**90-day remediation rate, n (%)**	20 (19)	5 (20)	6 (14)	9 (24)	0.6
**90-day readmission rate, n (%)**	8 (7.6)	1 (4.0)	3 (7.1)	2 (5.2)	0.7
**Follow-up (months), median (IQR)**	16.00 (6–24)	18.00 (9–24)	16.00 (6–24)	12.00 (3–18)	0.07
**Significant renal function loss at last follow-up, n (%)**	43 (41)	9 (36)	19 (45)	15 (39)	0.7

Legend: OR: operative room, cc: cubic centimeter. ^1^ Median (IQR); n/N (%). ^2^ Kruskal–Wallis rank-sum test; Fisher’s exact test; Pearson’s Chi-squared test. * Morphine equivalent.

## Data Availability

The data sets generated during and/or analyzed during the current study are available in the SinglePortEpic repository [https://docs.google.com/spreadsheets/d/1ESFUPYYaWXjKQyxTYypbSI2DRL5PyZSw/edit?usp=drive_link&ouid=102992091154524323243&rtpof=true&sd=true] (accessed on 23 January 2025).

## References

[1] Klatte T., Ficarra V., Gratzke C., Kaouk J., Kutikov A., Macchi V., Mottrie A., Porpiglia F., Porter J., Rogers C.G. (2015). A Literature Review of Renal Surgical Anatomy and Surgical Strategies for Partial Nephrectomy. Eur. Urol..

[2] Bertolo R., Ditonno F., Veccia A., De Marco V., Migliorini F., Porcaro A.B., Rizzetto R., Cerruto M.A., Autorino R., Antonelli A. (2024). Postoperative outcomes of transperitoneal versus retroperitoneal robotic partial nephrectomy: A propensity-score matched comparison focused on patient mobilization, return to bowel function, and pain. J. Robot. Surg..

[3] Porpiglia F., Mari A., Amparore D., Fiori C., Antonelli A., Artibani W., Bove P., Brunocilla E., Capitanio U., Da Pozzo L. (2021). Transperitoneal vs retroperitoneal minimally invasive partial nephrectomy: Comparison of perioperative outcomes and functional follow-up in a large multi-institutional cohort (The RECORD 2 Project). Surg. Endosc..

[4] Fu J., Ye S., Ye H. (2015). Retroperitoneal Versus Transperitoneal Laparoscopic Partial Nephrectomy: A Systematic Review Meta-analysis. Chin. Med. Sci. J..

[5] Arora S., Heulitt G., Menon M., Jeong W., Ahlawat R.K., Capitanio U., Moon D.A., Maes K.K., Rawal S., Mottrie A. (2018). Retroperitoneal vs Transperitoneal Robot-assisted Partial Nephrectomy: Comparison in a Multi-institutional Setting. Urology.

[6] Zillioux J.M., Krupski T.L. (2017). Patient positioning during minimally invasive surgery: What is current best practice?. Robot. Surg..

[7] Mertens zur Borg I.R.A.M., Lim A., Verbrugge S.J.C., IJzermans J.N.M., Klein J. (2004). Effect of intraabdominal pressure elevation and positioning on hemodynamic responses during carbon dioxide pneumoperitoneum for laparoscopic donor nephrectomy: A prospective controlled clinical study. Surg. Endosc..

[8] Mills J.T., Burris M.B., Warburton D.J., Conaway M.R., Schenkman N.S., Krupski T.L. (2013). Positioning injuries associated with robotic assisted urological surgery. J. Urol..

[9] Okhawere K.E., Beksac A.T., Wilson M.P., Korn T.G., Meilika K.N., Harrison R., Morgantini L.S., Ahmed M., Mehrazin R., Abaza R. (2022). A Propensity-Matched Comparison of the Perioperative Outcomes Between Single-Port and Multi-Port Robotic Assisted Partial Nephrectomy: A Report from the Single Port Advanced Research Consortium (SPARC). J. Endourol..

[10] Fan G., Wang J., Wang Y., Chen Y., Wu Y., Cai S., Li Y., Tang T. (2025). Comparative short-term efficacy and safety analysis of a single-port robot in nephrectomy. J. Robot. Surg..

[11] Pellegrino A.A., Chen G., Morgantini L., Calvo R.S., Crivellaro S. (2023). Simplifying Retroperitoneal Robotic Single-port Surgery: Novel Supine Anterior Retroperitoneal Access. Eur. Urol..

[12] Rohi A., Olofsson M.E.T., Jakobsson J.G. (2022). Ambulatory anesthesia and discharge: An update around guidelines and trends. Curr. Opin. Anaesthesiol..

[13] Campbell S.C., Uzzo R.G., Karam J.A., Chang S.S., Clark P.E., Souter L. (2021). Renal Mass and Localized Renal Cancer: Evaluation, Management, and Follow-up: AUA Guideline: Part II. J. Urol..

[14] Assel M., Sjoberg D., Elders A., Wang X., Huo D., Botchway A., Delfino K., Fan Y., Zhao Z., Koyama T. (2019). Guidelines for Reporting of Statistics for Clinical Research in Urology. Eur. Urol..

[15] Pandolfo S.D., Cerrato C., Wu Z., Franco A., Del Giudice F., Sciarra A., Verze P., Lucarelli G., Imbimbo C., Perdonà S. (2023). A systematic review of robot-assisted partial nephrectomy outcomes for advanced indications: Large tumors (cT2-T3), solitary kidney, completely endophytic, hilar, recurrent, and multiple renal tumors. Asian J. Urol..

[16] Ng A.M., Shah P.H., Kavoussi L.R. (2017). Laparoscopic Partial Nephrectomy: A Narrative Review and Comparison with Open and Robotic Partial Nephrectomy. J. Endourol..

[17] Calpin G.G., Ryan F.R., McHugh F.T., McGuire B.B. (2023). Comparing the outcomes of open, laparoscopic and robot-assisted partial nephrectomy: A network meta-analysis. BJU Int..

[18] Tameze Y., Low Y.H. (2022). Outpatient Robotic surgery: Considerations for the Anesthesiologist. Adv. Anesth..

[19] Nadu A., Ekstein P., Szold A., Friedman A., Nakache R., Cohen Y., Matzkin H., Weinbroum A.A. (2005). Ventilatory and hemodynamic changes during retroperitoneal and transperitoneal laparoscopic nephrectomy: A prospective real-time comparison. J. Urol..

[20] Peng C., Shen H., Cao S., Wu S., Huang Q., Li S., Li H.Z., Zhang X., Wang B., Cao J. (2023). Effects of Retroperitoneal or Transperitoneal Pneumoperitoneum on Inferior Vena Cava Hemodynamics and Cardiopulmonary Function: A Prospective Real-Time Comparison. J. Endourol..

[21] Garg M., Singh V., Sinha R.J., Sharma P. (2014). Prospective randomized comparison of transperitoneal vs retroperitoneal laparoscopic simple nephrectomy. Urology.

[22] Lambertini L., Pacini M., Morgantini L., Smith J., Torres-Anguiano J.R., Crivellaro S. (2024). The atlas of supine single port extraperitoneal access. Int. Braz. J. Urol..

[23] Jacobs C.J., Steyn W.H., Boon J.M. (2004). Segmental nerve damage during a McBurney’s incision: A cadaveric study. Surg. Radiol. Anat..

[24] Lambertini L., Pacini M., Calvo R.S., Anguiano J.R.T., Cannoletta D., Pettenuzzo G., DI Maida F., Valastro F., Mari A., Bignante G. (2025). Retroperitoneal single port vs. transperitoneal multiport robot assisted partial nephrectomy in patients with highly hostile abdomen: Comparative analysis from a tertiary care center. Minerva Urol. Nephrol..

